# Wrist malpractice claims in Northern Norway 2005–2014. Lessons to be learned

**DOI:** 10.1080/22423982.2018.1483690

**Published:** 2018-06-18

**Authors:** Jan Norum, Lise Balteskard, Mette Willumstad Thomsen, Hebe Desiree Kvernmo

**Affiliations:** a Department of Clinical Medicine, Medical Imaging Research Group, Faculty of Health Sciences, UiT- The Arctic University of Norway, Tromsø, Norway; b Department of Surgery, Finnmark hospital trust, Hammerfest, Norway; c Centre for Clinical Documentation and Evaluation, Northern Norway Regional Health Authority trust, Tromsø, Norway; d Department of Economics, Norwegian System of Patient Injury Compensation, Oslo, Norway; e Department of Orthopaedic, Hand and Plastic Surgery, University Hospital of North Norway, Tromsø, Norway

**Keywords:** Wrist surgery, complains, compensation, Northern Norway

## Abstract

Rough weather conditions in the subarctic areas of Norway may influence on the risk of wrist fracture. We implemented data from the Norwegian System of Patient Injury Compensation (NPE). All claims due to wrist surgery, performed at the public hospitals in Northern Norway, during 2005-2014 were analyzed. We employed the ICD-10 classification codes S52.5 (fracture of distal end of radius) and S52.6 (fracture of distal end of radius and ulna). Treatment was defined by NCSP codes. 84 patients (0.3%) complained. Females complained four times more often than males did (P = 0.005) and received five times more frequently a compensation (P < 0.001). NPE accepted 34 claims (40%) for injury compensation (0.1% of patients). The percentage of claims accepted for compensation decreased from 48% to 30% during study period, probably due to delay in filling claims. The main causes of complains were pain, reduced range of motion, malfunction and weakness (35/84). The main causes of compensation were “operative treatment should have been performed” (14/34) and “wrong operative method applied” (13/34). The mean amount per compensation was €14,927 (€0–€52,995). Stonger focus on quality of care, updated guidelines and shared decission-making may reduce the number og complains and compensations.

During the last decades, medical errors have received considerable attention. The financial and social consequences of such errors may be significant [1]. Recently, the Norwegian System of Patient Injury Compensation (NPE) released their 2015 annual report and statistics for the regional health authorities [2]. Orthopaedic surgery constituted 37% of all complains and 40% of them received a compensation. There were no difference in complains and acceptance rate of compensation between the four health regions (northern, central, western and south-eastern) of Norway. The frequency of complains and malpractice has worried the Norwegian Orthopaedic Association (NOA) [3]. From a circumpolar perspective, it would be of interest to clarify whether there are differences within the northern region. For example between areas more populated with Sami people (e.g. Finnmark County) and the others.

Distal radial fractures are the most common fractures in Norway and account for one fifth of all fractures [3]. Operative treatment has long been the treatment of choice for displaced, unstable fractures. Closed reduction with percutaneous pin fixation and/or external fixation was previously the most common treatment methods for unstable fractures [4], but today open reduction and volar locking plating is dominating.

Complications and patient injury in wrist surgery do exist and lessons may be learned. The incidence of complications in hand surgery has been reported ranging between 2.5% and 25% [4–8]. The most common complications in treatment of distal radius fractures have been loss of reduction, median nerve injury or compression, ligament injuries, surgical site infection and complex regional pain syndrome (CRPS). Complications may be due to individual mistakes or system failures. Individual mistakes may be due to incompetence, inattention, insufficient follow-up, unawareness of fracture instability, etc. To minimise complications and malpractice on a system level, health care administrators may run campaigns to improve staff’s attention during treatment, for example the “safe surgery campaign” [9,10]. Another and maybe more important measure is the effort to reduce the number of complications by improving surgeons’ expertise or skills [11] or by giving better training and supervision [12]. Pappas et al. [12] recommended that hand surgeons should stay updated within their field of surgery, know their department’s quality of care figures and be aware of the present national and international guidelines. Furthermore, they should stay focused on quality of care, learn from reported patient complications/injuries, keep patients informed and systematically document diagnosis, information and treatment in the electronic patient record (EPR) system.

During the last decades, investigators have documented an increasing number of medical malpractice claims [12]. Consequently, health care administrators worldwide have focused on preventive strategies and introduced a focus on quality of care to minimise the number of claims. So also in Northern Norway. Whereas this region covers almost half of Norway’s land mass and is about two thirds of the size of the UK, the population is only 480,740 inhabitants (as of 1 January 2015). Vast distances have been a constant challenge to the Northern Norway Regional Health Authority (NNRHA) trust in terms of quality of care, costs and logistics. During the last decade, a trend towards centralisation of complex cases has occurred both in Northern Norway and worldwide.

All Norwegian patients may claim compensation for malpractice experienced in the specialised health care. To qualify for compensation, the injury must have led to a financial loss. The NPE handle the requests and run a database including all claims received and compensations given. Despite many patients undergoing improper treatment do not complain, the NPE database may contain valuable quality of care information. In this study, we aimed to employ Northern Norwegian data on patient injury compensation for patients treated for distal radius fractures.

## Materials and methods

### Norwegian system of patient injury compensation (NPE)

The NPE, established in 1998, is a government agency subject to the Norwegian Ministry of Health and Care Services. It provides help and guidance to patients who have queries about their treatment and who plan to claim compensation for injuries. The claim should be due to an injury sustained during treatment at any hospital within the Norwegian public health care service. NPE takes care of the process and assists patients in how to complete the injury report form. They also inform patients about the processing of their claims. The main NPE financiers are the four regional health authority trusts. Furthermore, the Norwegian counties and the community health care cover a minor share. When a patient treated in the specialised health care receives a compensation, the local hospital has to cover the first €1,054 and 10% of the additional amount. The maximum hospital share is €10,540 per case.

### Patients with wrist fractures in Norwegian patient register (NPR)

NPR is a subsection of the Norwegian Directorate of Health (NDH). This register contains medical information on all patients visiting public hospitals or private hospitals with a reimbursement agreement with the regional health authorities. Only data from 2010 to 2014 was available for this study.

To indicate the percentage of patients claiming a compensation, we identified all patients in Northern Norway with a wrist fracture. We employed the diagnoses, according to the international classification of diseases (ICD-10), codes S52.5 (fracture of distal end of radius) and S52.6 (fracture of distal end of radius and ulna). When two fractures in the same patient were reported with more than 100 days in between, it was calculated as two separate fractures. When less than 100 days, it was concluded the same fracture undergoing control or retreatment.

### Patients complaining to NPE

We retrospectively analysed all complains to the NPE following orthopaedic wrist-fracture treatment performed at any of the public hospitals in Northern Norway during the 10-year period, 1 January 2005 and 31 December 2014. Furthermore, the following Nomesco Classification of Surgical Procedures (NCSP) procedure codes were employed; NCJ25 and NCJ 27 – external fixation, NCJ35 and NCJ37 – osteosynthesis using a bio-implant, NCJ45 and NCJ47 – osteosynthesis using wire, rod, cerclage or pin, NCJ55 or NCJ57 – osteosynthesis using intramedullary nail, NCJ65 and NCJ67 – osteosynthesis using plate and screws. There were in total 84 complains from 84 patients. The mean age was 57 years (range 8–84 years). Most of them were females (70 patients – 83%). Table 1 shows patient characteristics. When analysing compensations given, any accepted appeal on refusal was included. The status as of 1 June 2016 was employed.

**Table 1.** Characteristics of all patients treated, those claiming a compensation and those who received one.

		Total	Claims		Compensation given	
Characteristics		Numbers	Numbers	%	Numbers	%
Sex	Females	5,324*	70 (29*)	0.5*	29 (10*)	41 (34*)
	Males	2,922*	14 (4*)	0.1*	5 (1*)	36 (25*)
	Total	8,246*	84 (33*)	0.4*	34 (11*)	40 (33*)
Year of injury	2005	-	12		6	50
	2006		9		5	56
	2007		11		4	36
	2008		12		5	42
	2009		7		5	71
	2010	1,644	8	0.5	2	25
	2011	1,495	10	0.7	5	50
	2012	1,608	5	0.3	0	0
	2013	1,680	7	0.4	1	14
	2014	1,819	3	0.2	1	33
Hospital trust	Finnmark	1,075*	8 (4*)	0.4*	2	25
	UNN	3,404*	37 (12*)	0.4*	16	43
	Nordland	2,467*	24 (12*)	0.5*	9	38
	Helgeland	1,300*	15 (5*)	0.4*	7	47
County of residence	Finnmark		7		2	29
	Troms		29		12	41
	Nordland		44		19	43
	Others		3		0	0
	Not given		1		1	100
Diagnosis (ICD-10)	S52.5 S52.6	7380* 866*	78 (29*) 5 (2*)		30 (9*) 4 (1*)	38 (31*) 80 (50*)

*Data from 2010–2014.

### Statistical analysis and authorisation

NPE delivered the extracted information from their databank directly to the NNRHA in an anonymous version available on an Excel platform. Despite anonymous data, the file had an access code and the code was only available to the researchers. Consequently, none of the investigators had any access to patient identifiable data.

Centre of Clinical Documentation and Evaluation (SKDE) at the NNRHA trust has a concession from the Norwegian Data Protection Authority and confidentiality exemption from the Regional Committee for Medical and Health Research Ethics (REK) to provide access to unique personal data from the Norwegian Patient Registry (NPR). SKDE delivered 5 years data to the study in an anonymous version. All data were aggregated on patients’ county of residence and their treating hospital’s trust.

We employed the Microsoft Excel 2016 for the local database, calculations and statistical analyses. Furthermore, we used descriptive statistics and employed the Chi-square test for the comparison between subgroups, institutions and counties. Significance was set to 5%.

Compensations were given in Norwegian Krone (NOK) and converted into Euros (€) at a rate of €1=9.4839 as of 29 March 2016 (www.norges-bank.no).

As we imported anonymous data and focused on quality of care and health economics, no ethical committee or Data Inspectorate approval was necessary. Consequently, no approval from the Regional Committees for Medical and Health Research Ethics (REK) was necessary. Similarly, no approval from the Norwegian Social Science Data Services (NSD) was required.

## Results

### Injury compensation given

Compared with males, females complained four times more often (P<0.005) and received five times more frequently a compensation (P<0.001). Figures are shown in Table 1. During study period, the NPE accepted 34 out of 84 claims from patients (40%). Consequently, a mean of 3.4 patients got annually a compensation (range 0–6 injuries/year). There was a falling trend, as only two patients got a compensation during the past 3 years. Details are given in Table 1.

Looking at patients’ county of residence, Nordland and Troms had both eight persons per 100.000 inhabitants who received compensation. The figure of Finnmark county was only 3/100,000 inhabitants.

Focusing on hospital trust and the percentage of seekers given compensation, we revealed the following figures: University hospital of North-Norway (UNN) trust 43%, Nordland hospital trust 38%, Helgeland hospital trust 47% and Finnmark hospital trust 25%. The number of seekers and receivers of compensation decreased by one third between the two 5-year periods [2005–2009 and 2010–2014]. The percentage claiming for a compensation during the period 2010–2014 was similar in all three hospital trusts (range 0.4–0.5%). During the whole period, the number of patients seeking compensation dropped in the Helgeland and UNN hospital trusts and was stable in the Nordland and Finnmark hospital trusts.

The main causes of patient injury compensation were “operative treatment should have been performed” (14/34 patients) and “wrong operative methods used” (13/34 patients). The latter indicated lack of stable internal fixation in terms of plate and screws (5 cases), too tight and wrong plastering (5 cases) and unsatisfactory fracture reduction prior to fixation (2 cases). Looking at the subgroup “operative treatment should have been done”, nine patients should have undergone operative treatment and five received operative intervention too late during the care stream. Other failures were x-ray not taken during follow-up when exchanging the plastering (3 cases) and too long interval from treatment to follow up. In one case, the surgeon used too long screws and damaged a tendon of the thumb and one patient underwent an unnecessary operation (using plate and screws). Table 2 shows an overview of the causes.

**Table 2.** The causes for the acceptance of patient injury compensation.

Causes of injury compensation	Patients	%
Lack of documentation	1	3
Equipment failure	1	3
Treatment not indicated	1	3
Insufficient treatment	4	12
Wrong method used	13	38
Should have been operated	14	41
Total	34	100

The frequency of complains could be calculated for the latter 5-year period (2010–2014). There were in total 8,246 patients treated for fracture (Table 1) of the wrist and 33 complained (0.4%) and 11 (0.1%) of them got a compensation. Furthermore, persons seeking compensation were older than the total group treated. The mean age of the groups were 57 years (range 8–84 years) and 45 years (range 0–106 years), respectively.

### No injury compensation given

Fifty patients (60%) claiming injury compensation did not get any compensation at all. The majority of them (35 patients) complained of symptoms (pain, reduced range of motion, malfunction weakness) from the wrist. One complained of delayed diagnosis, three of complications due to infections, six argued that they should have undergone surgery and four argued that the plastering had been too tight. In all cases, the NPE could not reveal any correlation between symptoms and the treatment or concluded complains were normally expected side effects of treatment.

### Value of compensation

In 32 out of the 34 accepted cases for compensation, the case was closed and the NPE had concluded the amount of compensation. A total compensation of €477,662 (mean €14,927, range €0–€52,995) was concluded. In two cases, the amount was still under consideration and two cases were closed (no reason given in the database) without any compensation given. Furthermore, from the first period (2005–2009) to the latter (2010–2014), the cost per compensation dropped from €16,585 to €12,027. However, two cases during the latter period were not closed and the falling trend must therefore be handled with caution.

## Discussion

Very few patients complained after treatment of wrist fracture and 60% of cases were dismissed. The main causes for compensation were “wrong operative method(s) used” and “operative treatment should have been performed”. The mean amount of compensation per patient dropped during study period.

In total (all causes), there were annually more than 5,000 claims to the NPE [13,14].

Whereas females, during the period 2011–2015, constituted half (53%) of all complains to the NPE, this was not the situation for treatment of wrist fractures. Here we disclosed females complained five times more often than men did, and the seekers were generally older than the whole group treated. Based on the NPE data [13,14], wrist fracture constituted only about 1.7% of the total number of complains. Trauma surgery and orthopaedics have been reported the discipline most frequently confronted with claims of medical malpractice [15]. In a large study [5] including 10,646 patients, the overall incidence of complications within 30 days after hand surgery was only 2.5%. Older age, diabetes, chronic obstructive pulmonary disease, congestive heart failure, atherosclerosis, steroids, bleeding disorder, increasing wound class, emergency procedure, longer operative time and preoperative transfusion were associated with significantly higher risk of complications. Local anaesthesia and outpatient surgery were associated with lower risk. The most common complication was surgical-site infection.

Whereas complications are expected, few of them ended up in complains for compensation and the figures were falling in Norway. The latter may be due to an improved focus on quality of care, national campaigns for improved patient safety and national guidelines [i.e. the guidelines of the Norwegian Orthopaedic Association (NOA), www.wristfractures.no]. The clinicians themselves may also improve their results by knowing and abiding by the standard of care, keeping patients informed and developing good relationships with them, and meticulously documenting [12].

Looking at our results, the NPE frequently concluded that some of the complains had to be expected after surgery. When patients still complain, this may be due to an imbalance of information given and needed on possible long-lasting symptoms due to the fracture(s) and/or the treatment itself. We argue that the incorporation of shared decision-making into daily practise is essential in keeping malpractice complains to a minimum. This may be performed through high-quality oral and written information. The majority of cases for compensation was that operative treatment should have been performed. Gaspar and colleagues [16] argued that in the treatment of painful arthritic wrist, a detailed understanding of the risk factors was essential for surgeons so that patients may be counselled accordingly and that alternative treatment options may be considered. We argue that this is also the situation in the treatment and follow up of fractures in the hand and wrist.

The mean amount paid in compensation was low (€14,927) and it was reduced from the first to the latter 5-year period. However, this trend must be handled with caution. According to law regulations, the patient may claim compensation within a 3 years time following the injury/complication. Consequently, further injury claims for 2013 and 2014 may still occur. Similarly, some patients may still appeal the refusal of compensation and alter the total amount paid for the last years.

Matsen et al. [17] suggested that the incidence of claims due to wrist fractures and claims paid could be reduced if surgeons acquire and maintain the knowledge and skills necessary for the care of the common conditions they encounter, including fractures. In Norway, senior clinicians are regularly offered a four-month period every 5 year for professional upgrading/training and most of them take part in at least one course/international conference per year. In our opinion, employers may more strongly secure that the surgeons undergo programmes improving their skills during these periods or throughout the year by taking courses, etc.

Postoperative infection has been one of the complications in hand surgery [5]. The incidence of postoperative infections has been reported between 0.5% and 6.0%, depending on centre, type of surgery, and site of surgery [5,18,19]. Underestimated infections may complicate even minor injuries to the hand [20] and delayed interventions may cause serious damage and introduce costs. Reichert and colleagues [20] treated 172 in-patients in the period 1990–2000 because of this underestimation and calculated a total cost of 210,000 D-Mark would have been saved if adequate treatment had been initiated on time. We believe prophylactic interventions and keeping the patients and their general practitioners well informed about this risk is crucial in this setting.

Looking at patients’ county of residence, it was somewhat surprising that the number of complains per inhabitant in Finnmark (9.6/100.000 inhabitants) was half that of Troms and Nordland (18.5/100,000 inhabitants). Whereas the incidence of wrist fracture has been documented slightly lower in Finnmark compared with Troms and Nordland [21], the minor difference cannot explain this finding. In Norway, the age and gender adjusted rate during the period 2009–2014 was 244 wrist fractures per 100,000 inhabitants [21]. This is in accordance with Swedish and Finish figures [22,23].

It could be speculated why less patients complain in Finnmark County. Possible causes could be surgery that is more proper. However, differences in surgical techniques were minor between Finnmark, Troms and Nordland [21] and complex patients from Finnmark were referred to the regional centre at UNN Tromsø. Consequently, surgery that is more proper cannot explain the difference. Another cause may be due to cultural differences. Finnmark do have a higher proportion of Sami people, but we are not aware of any study documenting less complains among this group. Furthermore, the inhabitants of Finnmark have a lower level of education compared to Troms and Nordland. It could be speculated that patients with lower level of education experience the process of complaining more challenging than those with higher level of education. Consequently, less complains may be made. However, we did not reveal any studies or reports supporting this statement, but it should be explored in future studies.

In our study, 40% of patients in Northern Norway who claimed a compensation actually got it. National Norwegian data for 2005–2014 has not been published, but according to information from the NPE (Thomsen MW) the national figure was 43%. This indicates that the clinical practise in Northern Norway does not deviate from the national one.

In Northern Norway, advanced hand surgery (the most complicated cases) has been centralised by the NNRHA to the University hospital of North Norway, Tromsø. This is in accordance with the suggestions by a study group in the Netherlands [24]. They concluded that the majority of accepted claims (between 1993 and 2008) included treatment in the general surgery group. Consequently, they argued for hand injury treatment by adequately trained surgeons and preferably by a trained hand surgeon. We suggest that this should include the complex and complicated cases of wrist fractures.

## Conclusion

In summary, injury-compensation is rare, but wrist fracture is a common diagnosis at NPE.

Patients should be well informed about possible side effects or harms of treatment. Furthermore, we believe a constant focus on informed or shared decision-making may keep the compensation figures low. Similarly, national guidelines and organisational systems securing necessary competence and registries clarifying quality of care is mandatory. We argue that the injury compensation system, although it may represent only few of the actual cases, may be an important tool for the routinely monitoring of quality of care.
